# Liposome-encapsulated plasmid DNA of telomerase-specific oncolytic adenovirus with stealth effect on the immune system

**DOI:** 10.1038/s41598-017-14717-x

**Published:** 2017-10-26

**Authors:** Katsuyuki Aoyama, Shinji Kuroda, Toshiaki Morihiro, Nobuhiko Kanaya, Tetsushi Kubota, Yoshihiko Kakiuchi, Satoru Kikuchi, Masahiko Nishizaki, Shunsuke Kagawa, Hiroshi Tazawa, Toshiyoshi Fujiwara

**Affiliations:** 1 Department of Gastroenterological Surgery, Okayama University Graduate School of Medicine, Dentistry and Pharmaceutical Sciences, Okayama, Japan; 2 Center for Innovative Clinical Medicine, Okayama University Hospital, Okayama, Japan; 3 Minimally Invasive Therapy Center, Okayama University Hospital, Okayama, Japan

## Abstract

Oncolytic virotherapy has the disadvantage of being unsuitable for systemic delivery due to immune elimination. Liposomal encapsulation is well-recognized to reduce immune elimination and enhance the stability of drugs in the bloodstream. In the present study, the potential of liposome-encapsulated plasmid DNA of telomerase-specific oncolytic adenovirus (TelomeScan) expressing GFP (Lipo-pTS) as an oncolytic adenoviral agent suitable for systemic delivery was investigated. Lipo-pTS, which has a diameter of 40–50 nm, showed potent antitumor effects on HCT116 colon carcinoma cells *in vitro* and *in vivo*. Tumor selectivity of Lipo-pTS was independent of coxsackie and adenovirus receptor (CAR). Importantly, Lipo-pTS reduced production of adenovirus-neutralizing antibodies (AdNAbs) after intravenous administration into immune-competent mice compared to TelomeScan, and even in the presence of AdNAbs, Lipo-pTS maintained strong cytotoxicity. In conclusion, Lipo-pTS has the potential to become an oncolytic adenoviral agent suitable for systemic delivery with the characteristics of CAR-independent antitumor activity and a stealth effect on the immune system.

## Introduction

Oncolytic virotherapy is an emerging strategy for cancer treatment. Many oncolytic viruses have been developed from various types of viruses, such as adenovirus, herpes simplex virus, vaccinia virus, reovirus, poxvirus, and picornavirus, and some of them have been tested in clinical trials^1–3^. We previously established a telomerase-specific oncolytic adenovirus (Telomelysin), which is controlled to replicate selectively in tumor cells, not in normal cells, and induce oncolytic cell death^4^. After induction of cell death, progeny viruses are capable of spreading to surrounding tumor cells and cause continued oncolytic cell death. The safety of Telomelysin for humans was recently proven in the phase I clinical trial performed in the USA^5^. Based on its safety profile and promising preclinical data of combination therapy of Telomelysin and ionizing radiation, a phase I/II clinical trial is currently ongoing in Japan to evaluate the safety and efficacy of the combination^6^. While oncolytic adenoviruses have been proven to have promising antitumor effects, it is undeniably true that oncolytic adenovirotherapy has a critical disadvantage in that its application is basically limited to locoregional administration because of nonspecific trapping in the reticuloendothelial system (RES), such as the liver and spleen, and neutralization by the immune response after systemic administration^7^.

Application of nanotechnology to cancer treatment has progressed rapidly in recent years, resulting in a major contribution to the development of drug delivery systems^8^. Particle size is important for effective drug delivery, because larger particles tend to be captured by the RES, and smaller particles are easily eliminated by renal excretion^9,10^. Nanoparticles, which are defined as particles from 1 to 100 nm in size, can accumulate in tumor tissues more selectively through the enhanced permeability and retention (EPR) effect, which is characterized by the leakiness of tumor vasculature and the lack of an efficient lymphatic system in tumor tissues^11^. Among the many kinds of materials used for nanoparticles, liposomes are among the most common nanoparticles used as drug carriers; they encapsulate therapeutic agents such as proteins, peptides, and nucleotides inside the lipid bilayers and thus enhance their therapeutic efficacy by increasing their stability in the bloodstream^12^. Several liposomal pharmaceutical products have been approved for clinical application since liposomal doxorubicin, Doxil (Janssen, Titusville, NJ) first received US Food and Drug Administration (FDA) approval in 1995 for the treatment of AIDS-related Kaposi’s sarcoma^13^.

In terms of the development of oncolytic adenoviruses that can be administered systemically, although the idea of encapsulating oncolytic adenoviruses by liposome may be easily conceived, this strategy seems to have several issues. One is that encapsulated adenoviruses by liposome are likely to become too big (larger than 100 nm) to take advantage of the EPR effect, judged by the fact that an adenovirus itself has a diameter of 80 to 100 nm. Another is the issue of the nature of adenovirus infectivity; the original adenoviruses are internalized into cells through a multistep process that starts with attachment of the adenovirus capsids including the fiber proteins to the coxsackie and adenovirus receptor (CAR) on the cell surface. Liposome encapsulation of adenoviruses is likely to make this initial step unnecessary for virus infection.

To take advantage of both oncolytic adenoviruses having a strong tumor-specific antitumor effect and nanosized liposomes providing great stability in the bloodstream and tumor specificity by the EPR effect, we decided to use the plasmid DNA of telomerase-specific oncolytic adenovirus for liposomal encapsulation instead of adenovirus itself to reduce the nanoparticle size to the range in which the EPR effect is expected to work efficiently. In the present study, the potential of this liposome-encapsulated adenoviral plasmid DNA as a systemically-deliverable oncolytic adenovirus *in vitro* and *in vivo* was evaluated by examining the antitumor effect, CAR independency in virus infection, and the effect of the immune system, especially via neutralizing antibodies to adenovirus.

## Results

### Preparation and characterization of Lipo-pTS

**Figure 1:**
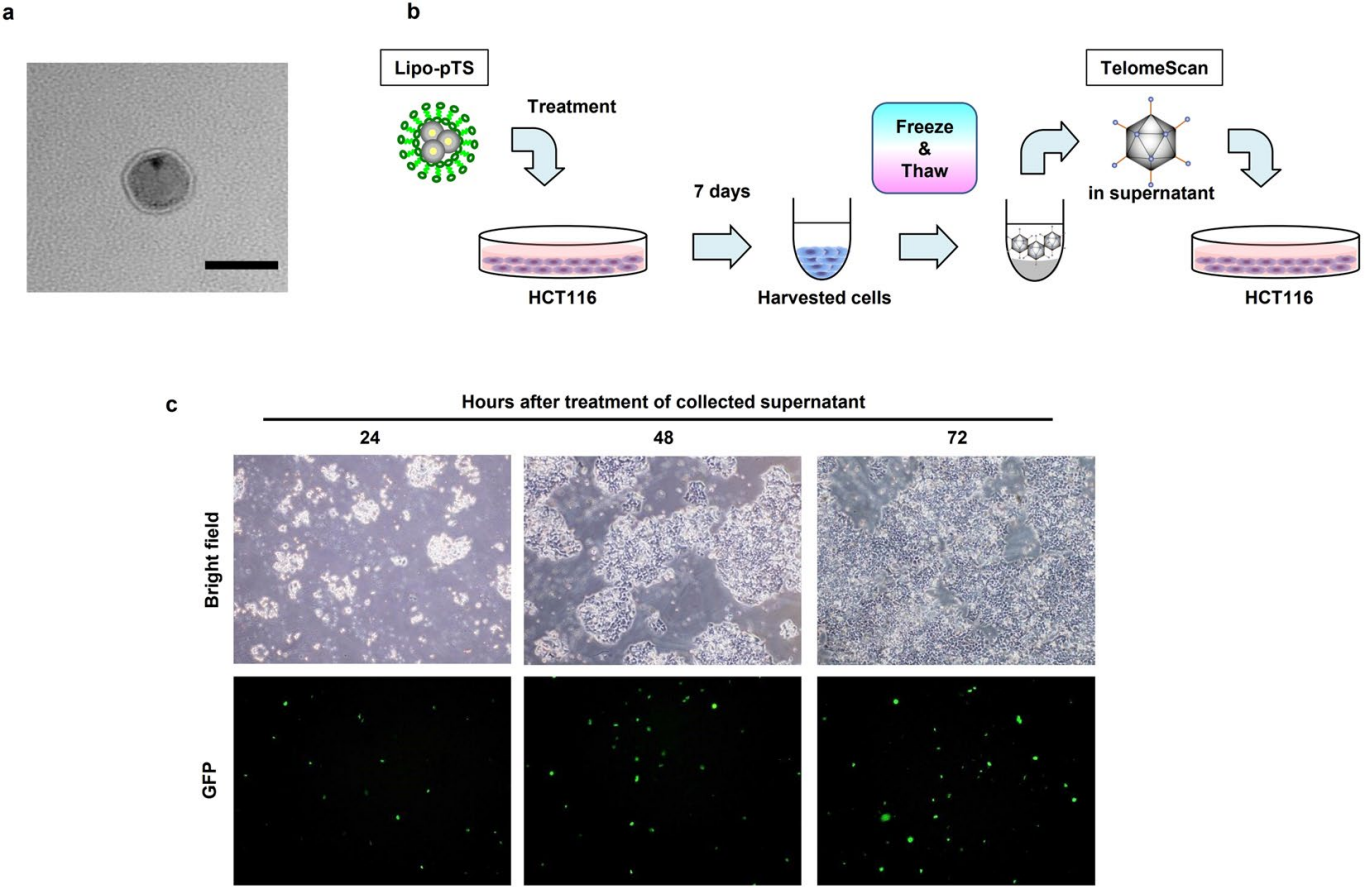
Characterization of Lipo-pTS. (**a**) Lipo-pTS seen on transmission electron microscopy. Scale bar, 50 nm. (**b**) The graphic shows the steps of the experiment to check the production of viable adenoviruses through the freeze-thaw process by which viable adenoviruses are isolated from the inside of cells. Briefly, HCT116 cells harvested at 7 days after Lipo-pTS treatment are subjected to a freeze-thaw process, and other HCT116 cells are treated with the supernatant collected by the freeze-thaw process. (**c**) HCT116 cells cultured for 24, 48, and 72 hours after the steps described in (**b**) are examined microscopically to check GFP expression. Note that GFP spots show successful production of viable oncolytic adenoviruses after Lipo-pTS treatment of HCT116 cells.

After amplification of TelomeScan plasmid DNAs, acquired plasmid DNAs were confirmed to be identical to the original TelomeScan plasmid DNAs based on genome size by electrophoresis. Lipo-pTS was observed as a 40–50-nm particle on transmission electron microscopy (Fig. 1a), which is considered a satisfactory size to benefit from the EPR effect^14^. To show that Lipo-pTS actually produces viable oncolytic adenoviral progenies in cancer cells after treatment, HCT116 cells harvested at 7 days after Lipo-pTS treatment were subjected to a freeze-thaw procedure (Fig. 1b). When other HCT116 cells were treated with the supernatant collected after 3 cycles of the freeze-thaw procedure, some GFP spots were observed at 24 hours after treatment on fluorescence microscopy, and they increased over time (Fig. 1c). These findings suggested that Lipo-pTS treatment has the potential to benefit from nanotechnology in terms of the particle size and produce actual viable adenoviruses that can cause further tumor-specific oncolytic cell death after subsequent infection to surrounding tumor cells.

### Cytotoxic efficacy of Lipo-pTS *in vitro* and *in vivo*

**Figure 2:**
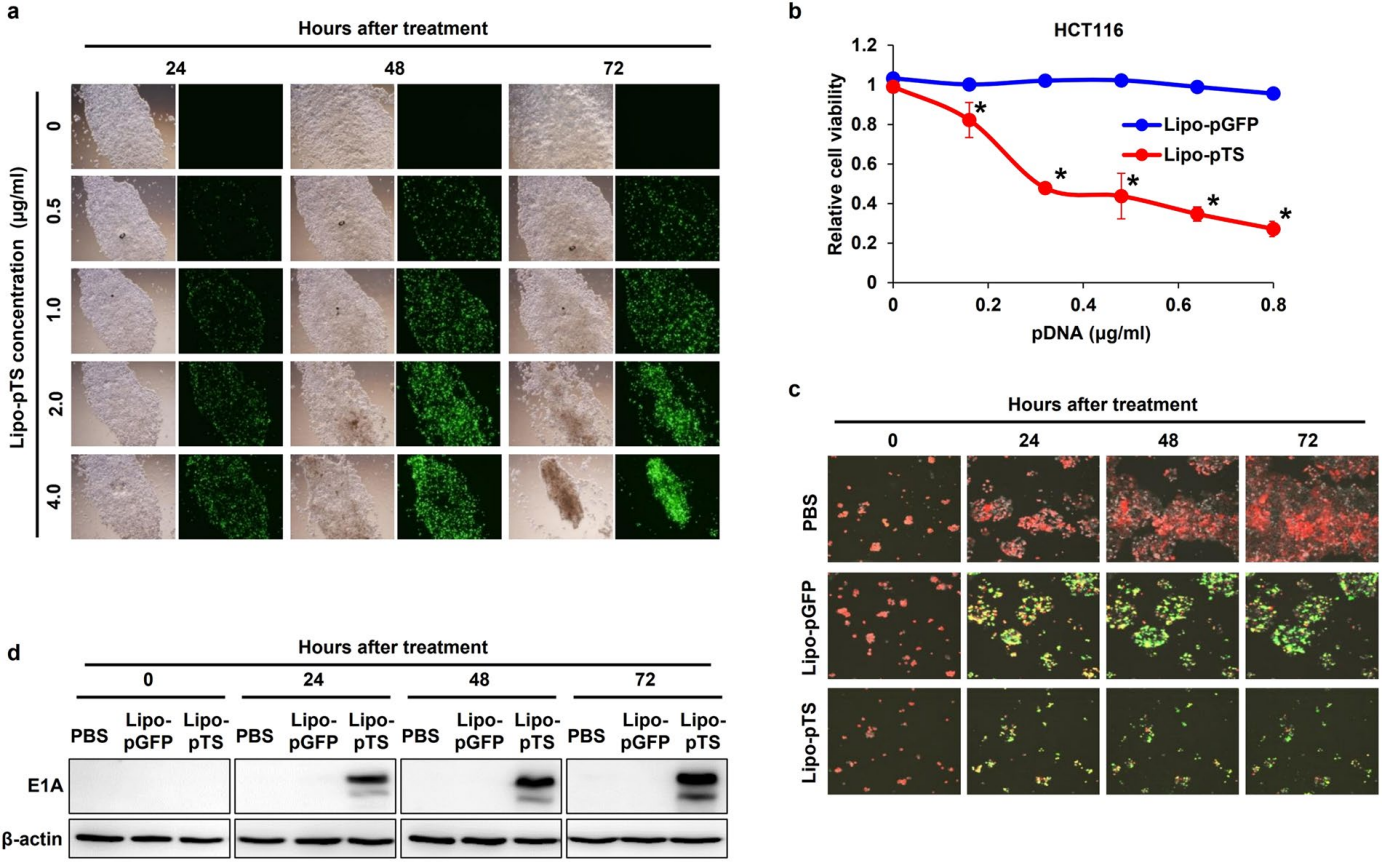
Infectivity and cytotoxicity of Lipo-pTS. (**a**) HCT116 cells are treated with different concentrations of Lipo-pTS for 24, 48, or 72 hours, and the infectivity, shown as GFP spots, is observed on fluorescence microscopy. (**b**) HCT116 cells are treated with several concentrations of Lipo-pTS or Lipo-pGFP for 3 days, and cell viability is assessed by the XTT assay. The percentage of viable cells relative to liposome-treated cells at each concentration is plotted. Error bars indicate 95% confidence intervals. *p < 0.01. (**c**) HCT116-RFP cells (red) are treated with Lipo-pTS, Lipo-pGFP, or PBS, and time-lapse images are taken at each 60 minutes for 3 days. Images at 0, 24, 48, and 72 hours are shown. (**d**) Western blot analysis of adenovirus type 5 E1A protein in whole cell lysates of HCT116 cells collected at 24, 48 or 72 hours after treatment with PBS, Lipo-pGFP or Lipo-pTS. The images were cropped from different blots run under the same experimental conditions. The original blots were presented in Supplementary Fig. 3.

To first examine the infectivity of Lipo-pTS, HCT116 cells (5.0 × 10^5^ cells/well in a 12-well plate) were treated with different concentrations (0, 0.5, 1.0, 2.0, 4.0 μg/ml) of Lipo-pTS for 24, 48, and 72 hours, and GFP expression was observed on fluorescence microscopy. GFP expression increased in a dose-dependent manner up to 2.0 μg/ml at 24, 48, and 72 hours after treatment, although no obvious increase in GFP expression at 4.0 μg/ml was observed at any time point (Fig. 2a). HCT116 cells treated with 2.0 or 4.0 μg/ml of Lipo-pTS were also found to be dying at 48 or 72 hours after treatment. The infectivity of Lipo-pTS on other cell lines was also confirmed by observation of GFP expression (Supplementary Fig. 1). Then, the cytotoxic efficacy of Lipo-pTS was tested *in vitro* by the XTT assay on HCT116 cells (1.0 × 10^3^ cells/well in a 96-well plate). The dose of Lipo-pTS was adjusted from 0 to 0.8 μg/ml according to the number of cells in this experiment, while 0 to 4.0 μg/ml of Lipo-pTS were used in the previous experiment using a 12-well plate. In this setting, Lipo-pTS significantly inhibited cell viability in a dose-dependent manner at 72 hours after treatment compared to Lipo-pGFP, and the half maximal inhibitory concentration (IC50) was approximately 0.4 μg/ml (Fig. 2b). This inhibitory effect was also observed on time-lapse imaging using HCT116-RFP cells, which showed that Lipo-pTS dramatically inhibited cell proliferation at the same time as expressing GFP, while Lipo-pGFP expressed GFP and suppressed cell growth very mildly (Fig. 2c, also see the Supplementary Video). In western blot analysis, expression of adenovirus type 5 protein was observed at 24 hours after Lipo-pTS treatment and increased over time up to 72 hours, which supported actual viral production after Lipo-pTS treatment, leading to oncolytic cell death (Fig. 2d).

**Figure 3:**
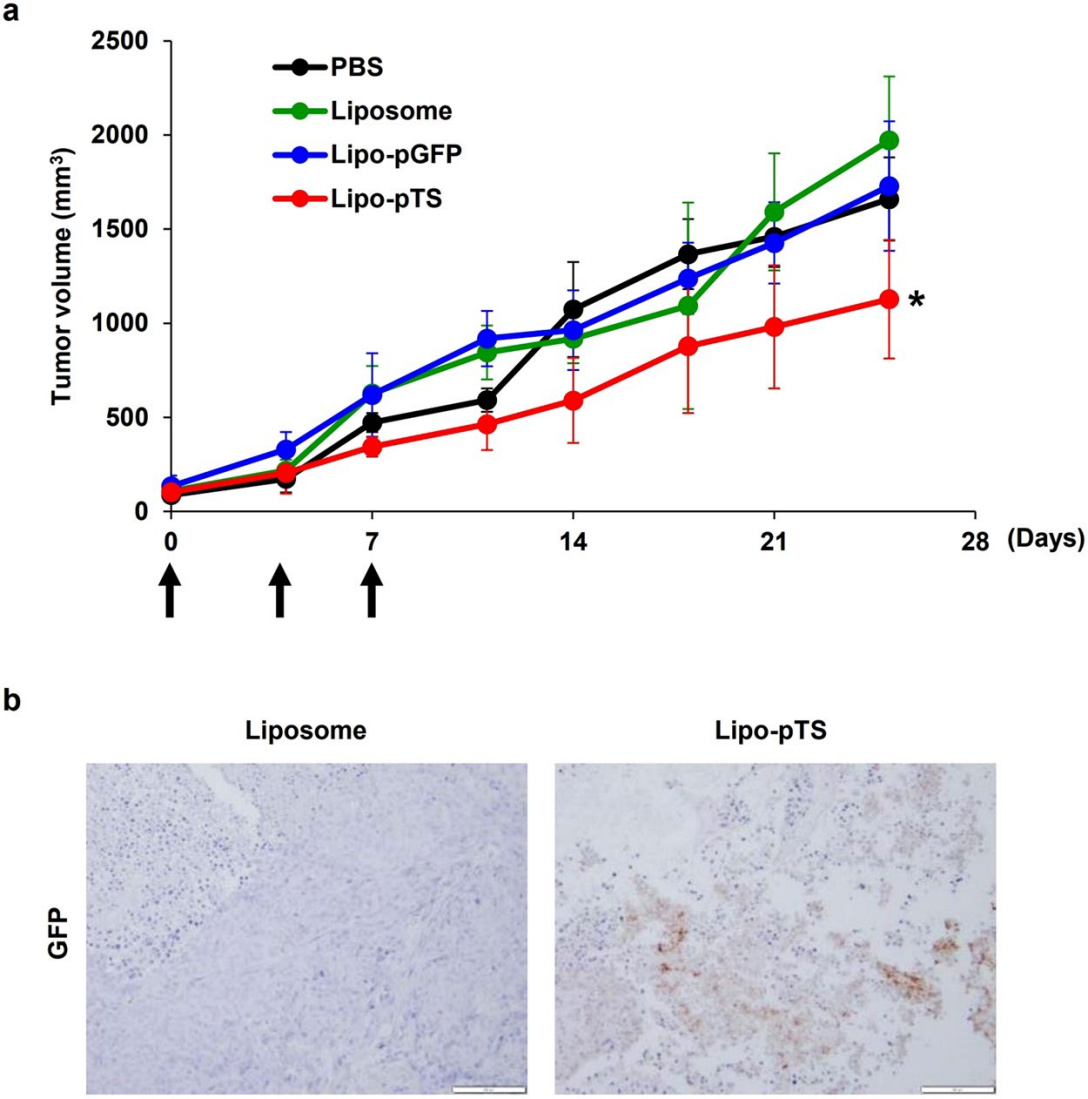
Antitumor effect of Lipo-pTS in the subcutaneous tumor mouse model. (**a**) HCT 116 tumors subcutaneously-implanted into BALB/c nude mice are treated with intratumoral injection of PBS, Liposome, Lipo-pGFP, or Lipo-pTS 3 times in a week. Tumor volume is measured twice a week until day 25. *p < 0.05. (**b**) GFP expression is examined with immunohistochemical staining of the tumor tissues harvested 2 days after the last (3rd) treatment by Liposome or Lipo-pTS.

In the *in vivo* model, when mice bearing subcutaneously-implanted HCT116 tumors were treated with PBS, liposome, Lipo-pGFP, or Lipo-pTS intratumorally 3 times in a week, Lipo-pTS inhibited tumor growth significantly more than the other control treatments (Fig. 3a). In another experimental setting in which subcutaneous tumors were harvested at 2 days after the 3rd treatment, immunohistochemical staining for GFP confirmed actual viral production in the tumor tissues after Lipo-pTS treatment (Fig. 3b). These findings demonstrated that Lipo-pTS can produce reasonable antitumor effects by producing viral progenies in the *in vitro* and *in vivo* models.

### CAR-independency of Lipo-pTS

**Figure 4:**
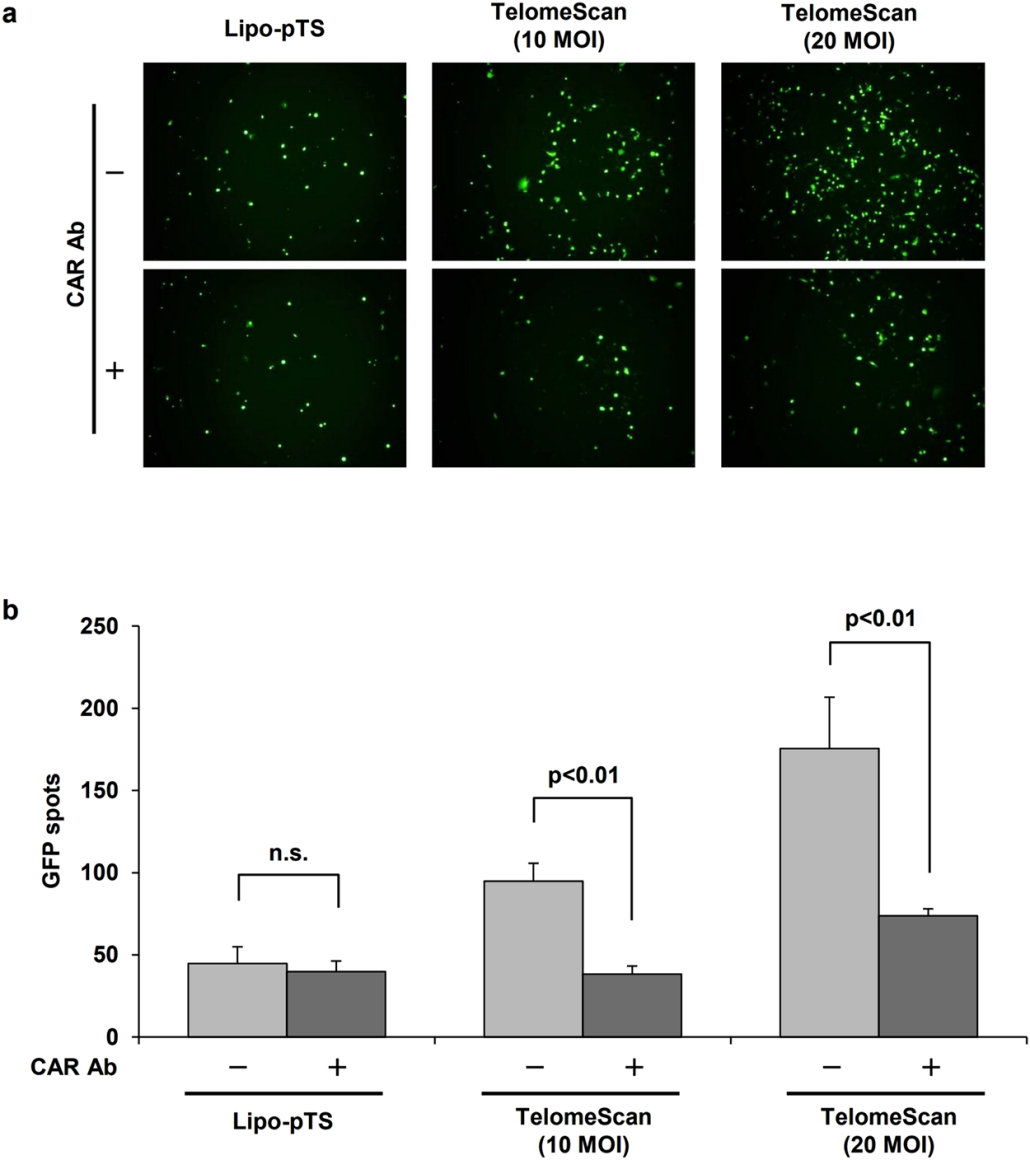
CAR-independency of Lipo-pTS. (**a**) HCT116 cells are treated with Lipo-pTS or TelomeScan with or without anti-CAR antibody pretreatment for 3 days, and GFP spots are observed on fluorescence microscopy. (**b**) The number of GFP spots is counted.

Since the infectivity of oncolytic adenoviral agents including TelomeScan, which are derived from a human adenovirus type 5, is basically depending on the CAR expression of cells, the original TelomeScan is not capable of having adequate cytotoxic effects against cancer cells whose CAR expression level is low^15^. In fact, the infectivity of TelomeScan (10 or 20 MOI) for HCT116 cells was dramatically inhibited by approximately 60% after addition of anti-CAR antibodies in the culture medium (Fig. 4a,b). On the other hand, almost no change was observed in infectivity of Lipo-pTS by addition of anti-CAR antibodies to the culture medium. These results showed that Lipo-pTS has the potential to infect and produce cytotoxic activity even on cancer cells that express little CAR on the cell surface because of its CAR-independency in infectivity, unlike the original TelomeScan.

### Stealth effect of Lipo-pTS on the immune system

**Figure 5:**
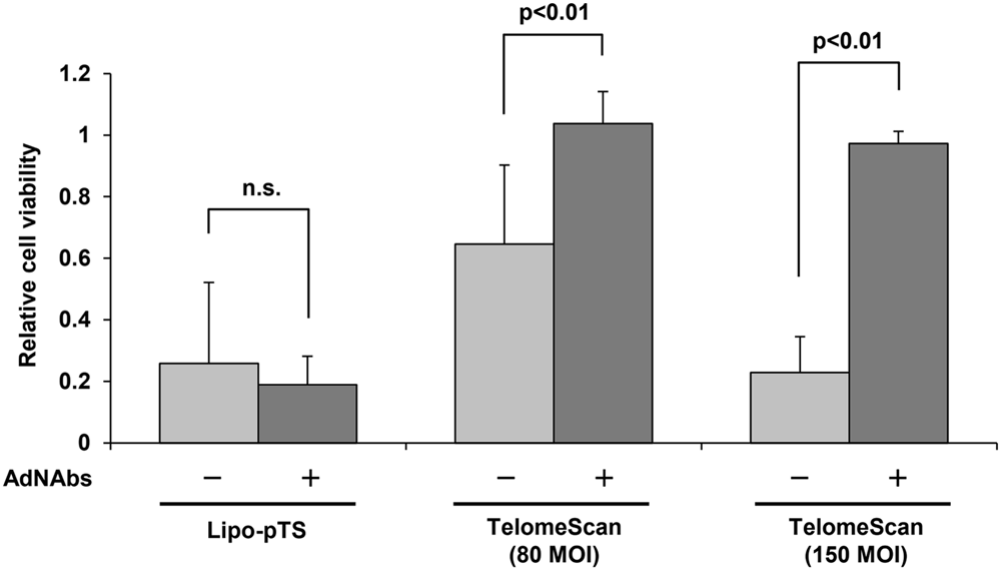
The stealth effect of Lipo-pTS providing protection from adenovirus neutralizing antibodies *in vitro*. HCT116 cells are treated with Lipo-pTS or TelomeScan in the presence or absence of adenovirus neutralizing antibodies for 3 days, and cell viability is examined by the XTT assay.

Another critical limitation of oncolytic adenoviruses derived from a human adenovirus type 5 is that they cannot be given systemically. One of the reasons for this is immune elimination via neutralizing antibodies to adenovirus type 5 (AdNAbs). Most adults have immunity to adenovirus type 5 because this is a kind of virus that causes the common cold. On *in vitro* assay, the infectivity and cytotoxicity of TelomeScan were actually suppressed strongly in the presence of AdNAbs (Fig. 5).

**Figure 6:**
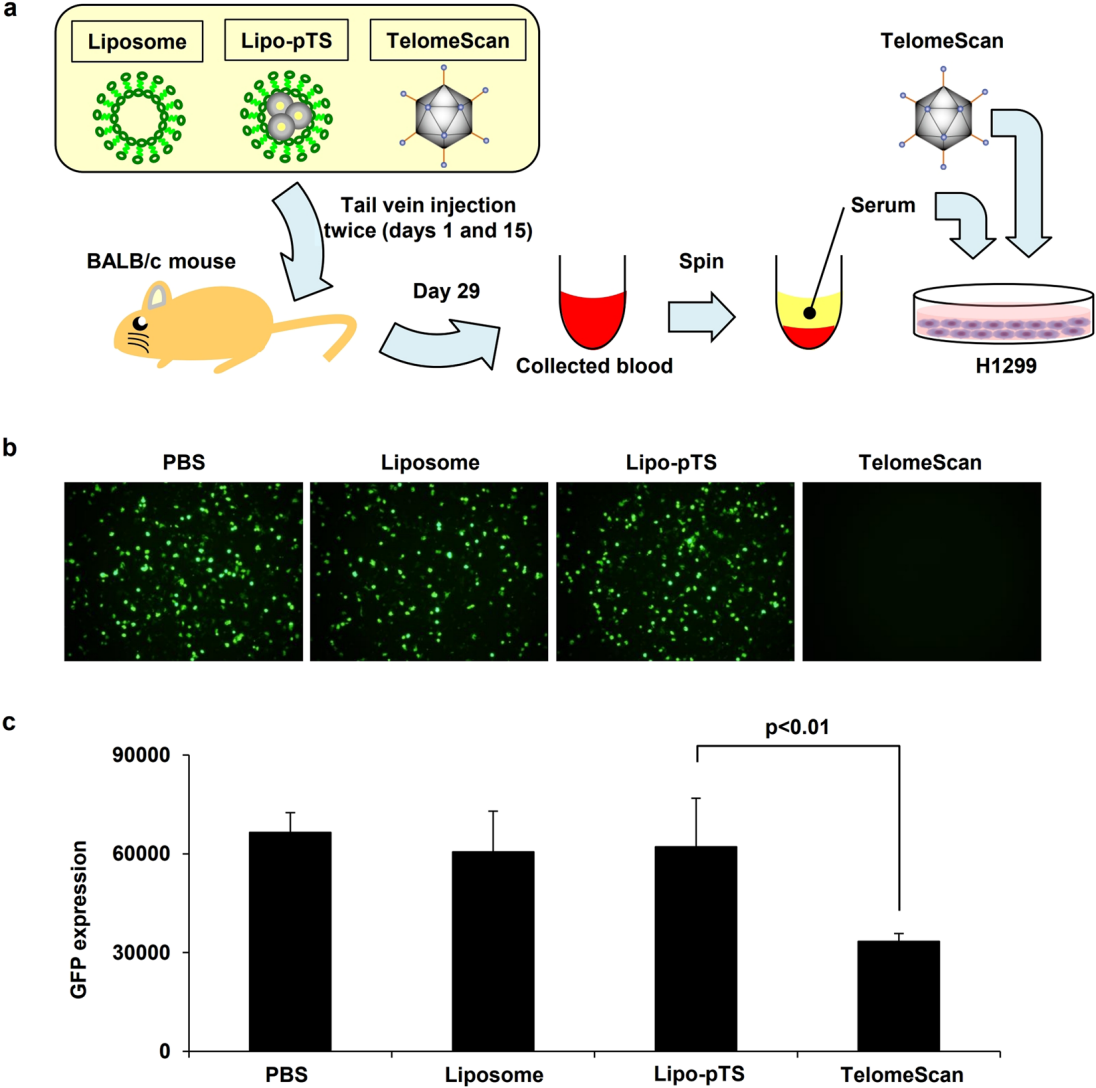
Stealth effect of Lipo-pTS providing protection from the immune system *in vivo*. (**a**) The graphic shows the steps of the experiment. Briefly, PBS, Liposome, Lipo-pTS, or TelomeScan is systemically injected into immune-competent BALB/c mice twice every 2 weeks (days 1 and 15), and blood is collected from each mouse at day 29. H1299 cells are treated with TelomeScan for 3 days in the presence of the serum collected from the mice. (**b**) After the steps described in (**a**), GFP spots are observed on fluorescence microscopy. (**c**) The intensity of GFP spots is measured by flow cytometry.

However, Lipo-pTS impressively showed as strong a cytotoxic effect even in the presence of AdNAbs as in the absence of AdNAbs, while the cytotoxic effect of TelomeScan (150 MOI) was almost nullified by AdNAbs, even though the cytotoxic effects of Lipo-pTS and TelomeScan (150 MOI) were almost the same in the absence of AdNAbs. Next, in the experiment to examine the production of AdNAbs after systemic administration into immune-competent mice (Fig. 6a), TelomeScan infectivity for H1299 cells was not impeded by the serum collected from the mice treated twice systemically with Lipo-pTS (10 μg), while TelomeScan infectivity was dramatically reduced by co-culture with the serum collected from the mice treated twice with the original TelomeScan (1.0 × 10^9^ PFU/time), which suggested that systemic administration of TelomeScan caused the production of AdNAbs in immune-competent mice, whereas Lipo-pTS did not (Fig. 6b,c). These findings proved that Lipo-pTS possesses a stealth effect on the immune system in the *in vivo* environment, meaning that systemic delivery of Lipo-pTS may be possible.

## Discussion

Oncolytic virotherapy has the potential to become the next breakthrough in cancer therapy following immunotherapy using immune checkpoint inhibitors. Oncolytic viruses have been developed worldwide since the 1990s based on the traits of strong cytotoxicity that viruses originally possess and ideal tumor specificity, which comes from appropriate genetic modification, and finally, the first oncolytic viral agent, named talimogene laherparepvec (T-VEC), was approved by the US Food and Drug Administration (FDA) in 2015 for the treatment of melanoma based on a successful phase III clinical trial in which T-VEC intratumoral injection decreased in size by ≥50 % not only 64% of injected lesions, but also 34% of uninjected non-visceral lesions and even 15% of visceral lesions, which means that local injection of oncolytic viruses interestingly induced systemic immunotherapeutic effects against melanoma, in addition to direct cytotoxic effects on the local lesions^16^. While this systemic effect by local injection of viruses is very attractive, local injection is sometimes demanding and challenging, especially for visceral tumors. Therefore, development of oncolytic viruses that can be delivered by systemic administration will still be required to expand the indications of oncolytic virotherapy. To overcome the unsuitability for systemic delivery, escape abilities from nonspecific exclusion by the RES, such as the liver, and from specific viral elimination by the immune system via neutralizing antibodies are needed, leading to enhancement of stability in the bloodstream, and one of the solutions is use of nanomaterials as a drug carrier.

Many kinds of nanomaterials, such as liposomes, micelles, polymers, and metal nanoparticles, have been developed and have contributed greatly to the development of drug delivery systems^17,18^. In addition to the above-mentioned Doxil, Abraxane (Celgene, Summit, NJ) is another nanotechnology-based chemotherapeutic agent for malignant tumors, which is a nanoparticle albumin-bound paclitaxel approved for breast cancer, non-small cell lung cancer, and pancreatic cancer by the FDA and for gastric cancer in Japan^19,20^. As for the use of nanoparticles in gene delivery, many types of nanoparticles have been developed as carriers of nucleic acids such as DNA, mRNA, siRNA, and miRNA, and some of the lipid-based and polymer-based nanoparticle-mediated gene therapies are currently being tested in clinical trials, though none of them has yet become commercially available^21,22^. Liposome, which is composed of physiological lipids well tolerated by the human body, is one of the most popular nanoparticles used as a drug carrier and has a stealth effect in the *in vivo* environment by reducing the immunogenicity of the drug-lipid complexes^12,23^. There are some previous reports in which encapsulation of an oncolytic virus itself by lipid was tested and showed some potential for use in systemic delivery^24,25^. However, this strategy seems to remain problematic in terms of an effective drug delivery system. The complex of oncolytic virus and lipid inevitably ends up being larger than 100 nm, which cannot benefit from the EPR effect, because the diameters of the adenovirus and the herpes simplex virus are approximately 80 nm and 100 nm, respectively. To solve this problem, we decided to encapsulate plasmid DNA of telomerase-specific oncolytic adenovirus instead of the adenovirus itself with liposome, and we successfully created nano-sized Lipo-pTS, which was shown by transmission electron microscopy to be reduced to 40–50 nm in size by eliminating the adenoviral capsids including the fibers. There is just one previous report in which a similar approach was tested, and it showed that systemic delivery of oncolytic viral genome DNA with liposomes can be a powerful alternative to naked adenovirus and can overcome the limited clinical applicability of conventional oncolytic adenoviruses^26^.

Oncolytic virotherapy is based on the concept that the viral genome is replicated in a host cell after viral infection and produces viral progenies that cause a unique type of cell death, called oncolytic cell death, followed by spread to surrounding cells. As a first step in our strategy, it was necessary to ensure that viral progenies that possess oncolytic ability were successfully produced in cancer cells after treatment by adenoviral plasmid DNA instead of adenovirus itself, and this phenomenon was clearly confirmed by the freeze-thaw cycle experiment. As a second step, Lipo-pTS showed a potent cytotoxic effect on cancer cells in *in vitro* and *in vivo* experiments compared to Lipo-pGFP, which is a complex of lipid and plasmid DNA expressing GFP, not having oncolytic ability, which proved that Lipo-pTS killed cells by an oncolytic effect, not just by the stress of gene transfection and GFP expression. Oncolytic effect by Lipo-pTS seemed to be observed within 24 hours after treatment based on the time-lapse imaging (Fig. 2c), and this may be supported by the previous report which showed that viral copy number of TelomeScan was amplified more than 10^5^-fold within 24 hours after infection^27^. Then, importantly, Lipo-pTS showed a stealth effect on the immune system in an *in vivo* experiment, in which systemic administration of Lipo-pTS into immune-competent mice did not produce as much AdNAbs as TelomeScan injection. Moreover, Lipo-pTS produced a potent cytotoxic effect even in the presence of AdNAbs, which is very important, because most adults already have AdNAbs by acquired immunity, although the cytotoxicity of TelomeScan was almost nullified in the presence of AdNAbs. Adenovirus usually infects cells through contact with CAR via adenoviral fibers, leading to clathrin-mediated endocytosis^28^. This CAR-dependency, being undoubtedly useful for adenovirus in efficient infectivity, brings a limitation in oncolytic adenovirotherapy in that oncolytic adenovirus cannot be expected to produce an adequate therapeutic effect in cancer cells that do not express CAR on the surface. Our strategy, however, can overcome this limitation theoretically, and Lipo-pTS actually showed CAR-independency in the *in vitro* study, in which infectivity of Lipo-pTS was not affected by pretreatment with CAR antibodies, while infectivity of TelomeScan was dramatically reduced by approximately 60%. Moreover, given that TelomeScan, which expresses GFP, has also been developed as a diagnostic tool for malignant tumors, Lipo-pTS may also be useful as a diagnostic tool that can detect even metastatic sites and circulating tumor cells that express little CAR on the cell surface.

While this study showed the promising potential of liposome-encapsulated oncolytic adenovirus as a systemically-deliverable agent for cancer therapy, this study definitely has several limitations, such as no animal experiments with systemic administration, use of crude plasmid DNA of TelomeScan, and use of unmodified marketed liposomal agent. Therefore, further improvement and investigation should be attempted to develop a more efficient oncolytic adenoviral agent. As for the possibility of improvements on viral DNA, linearized plasmid DNA of oncolytic adenovirus by restriction enzyme, such as Pac I, in combination with reducible polymers reportedly produced a stronger cytotoxic effect than circular plasmid DNA, which may suggest that linearized DNA of oncolytic adenovirus could be better for effective viral replication in transfected cells^29^. Another meaningful improvement will be surface modification of nanoparticles. Attachment of polyethylene glycol, one of the most common modifications, can improve the pharmacokinetics of therapeutic agents by increasing their stability in blood circulation and reducing renal clearance, as well as decreasing immunogenicity^30,31^. Attachment of ligands, antibodies, or peptides that bind to specific receptors or proteins is another approach to improve drug efficacy by increasing tumor selectivity and facilitating drug uptake. Liposomes conjugated with cetuximab or trastuzumab, monoclonal antibodies targeting the epidermal growth factor receptor (EGFR) or the human epidermal growth factor receptor 2 (HER2), respectively, reportedly enhanced the cytotoxicity of chemotherapeutic agents encapsulated in liposomes by increasing drug accumulation in targeted tumor tissues^32–34^.

**Figure 7:**
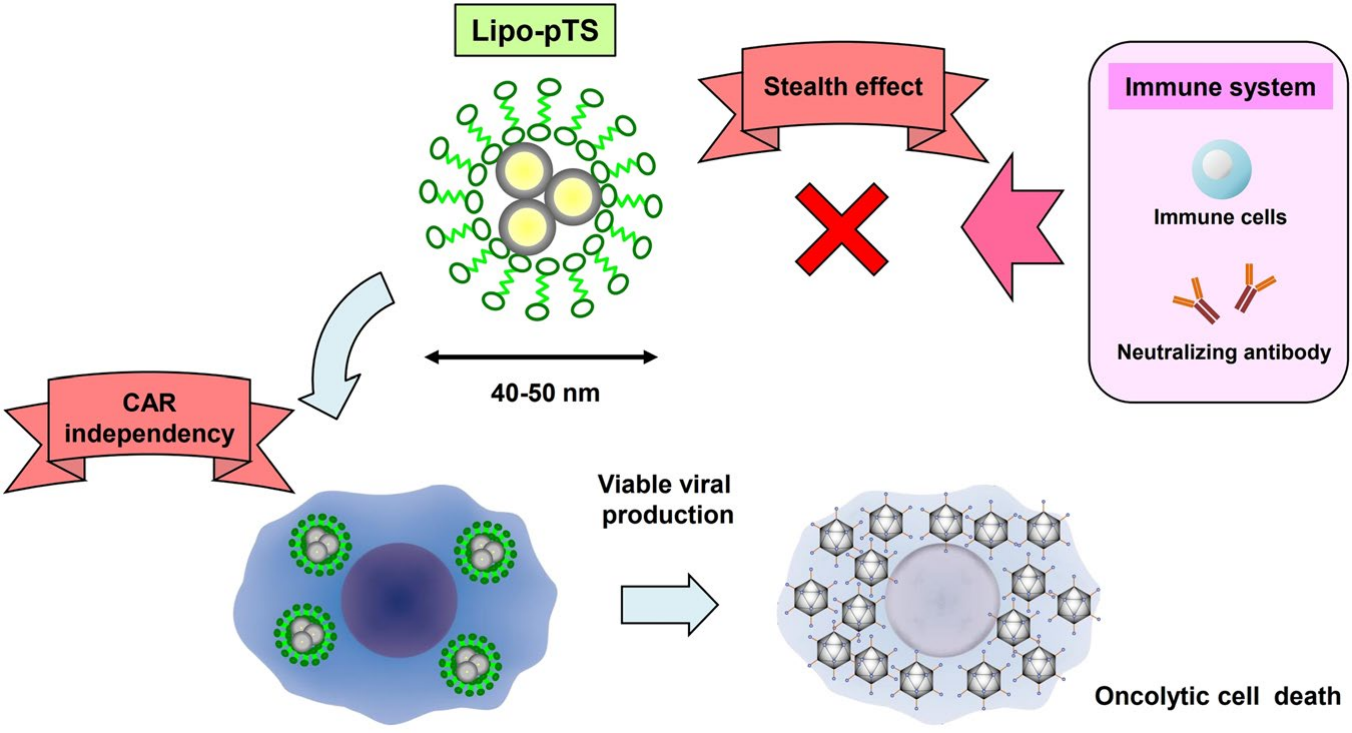
Schematic illustration.

In the present study, the strategy of encapsulating plasmid DNA of oncolytic adenovirus with liposome was found to have the potential to maximize the advantages of nanotechnology in medicine and overcome the disadvantages of oncolytic virotherapy through the results that Lipo-pTS, possessing CAR-independency and a stealth effect on the immune system, displayed a potent cytotoxic effect on cancer cells *in vitro* and *in vivo* by producing viable oncolytic adenoviruses (Fig. 7). Although further examination and further modification are definitely necessary, this study will hopefully serve as a foundation for development of systemically-deliverable oncolytic viral agents.

## Materials and Methods

### Cell lines and materials

HCT116, a human colon carcinoma cell line and HCT116-expressing red fluorescent protein (RFP) (HCT116-RFP) were used in this study. HCT116 cells were purchased from American Type Culture Collection (ATCC, Manassas, VA) and cultured in McCoy’s 5A (Modified) Medium (Life Technology, Carlsbad, CA) supplemented with 10% fetal bovine serum and 1% penicillin–streptomycin (100 U/ml). HCT116-RFP cells were obtained from AntiCancer, Inc. (San Diego, CA) and cultured in RPMI1640 medium (Sigma-Aldrich, Dorset, UK) supplemented with 10% fetal bovine serum and 1% penicillin–streptomycin (100 U/ml). Lipofectamine LTX and Plus Reagent (Life Technology) were used for liposomal encapsulation.

### Characterization and preparation of liposome-encapsulated telomerase-specific oncolytic adenoviral plasmid DNAs

We previously constructed telomerase-specific oncolytic adenoviruses expressing green fluorescent protein (GFP), TelomeScan, for monitoring viral replication following development of Telomelysin^35,36^. The construct of TelomeScan plasmid DNA was shown in Supplementary Fig. 2. TelomeScan plasmid DNAs were amplified in *E. coli* DH5α competent cells and purified using a Hispeed Plasmid Midi Kit (QIAGEN, Hilden, Germany) according to the manufacturer’s instructions. DNA concentration was measured by NanoDrop spectrophotometer (NanoDrop Technologies, Wilmington, DE) at 260 nm. TelomeScan plasmid DNA-lipid complexes (Lipo-pTS) were prepared using the amount of plasmid DNA and Lipofectamine recommended by the manufacturer. Complexes of lipid and TurboGFP plasmid DNA (Evrogen, Moscow, Russia) (Lipo-pGFP) were also prepared as a control agent. The dose and the concentration of DNA, not including liposome, were described as μg and μg/ml, respectively.

### Cell viability assay

HCT116 cells (1.0 × 10^3^ cells/well in a 96-well plate) (n = 5) were treated with various concentrations of Lipo-pTS, Lipo-pGFP, or liposome alone, and cell viability was determined at 3 days after treatment with a Cell Proliferation Kit II (XTT) (Roche) according to the manufacturer’s protocol. The viability of liposome-treated cells was considered 100%. Time-lapse images were taken with a confocal laser scanning microscope (Fluoview FV10i) (OLYMPUS, Tokyo, Japan) every 60 minutes for 3 days after treatment with Lipo-pTS, Lipo-pGFP, or PBS.

### Western blot analysis

Proteins extracted from whole-cell lysates were electrophoresed on 10 % SDS–polyacrylamide gels and were transferred to Hybond-polyvinylidene difluoride transfer membranes (GE Healthcare UK Ltd.). The membranes were incubated with primary antibodies against adenovirus type 5 E1A (BD Biosciences, Franklin Lakes, NJ) and β-actin (Sigma-Aldrich), followed by peroxidase-linked secondary antibody. The Amersham ECL chemiluminescence system (GE Healthcare UK Ltd.) was used to detect the peroxidase activity of the bound antibody. Equal loading of samples was confirmed by β-actin analysis.

### *In vivo* subcutaneous tumor model

HCT116 cells (2.0 × 10^6^ cells/mouse) were injected subcutaneously into the flanks of 5- to 6-week-old female BALB/c nude mice. When tumors reached a diameter of around 5–10 mm, the mice were divided into 4 groups (PBS, Liposome, Lipo-pGFP, and Lipo-pTS) (n = 7) and treated intratumorally 3 times (days 0, 4, and 7) with Lipo-pTS (10 μg), Lipo-pGFP (10 μg), liposome, or PBS. The perpendicular diameter of each tumor was measured twice a week until day 24, and tumor volume was calculated with the following formula: tumor volume (mm^3^) = *a* × *b* ^2^ × 0.5, where *a* is the longest diameter, *b* is the shortest diameter, and 0.5 is a constant to calculate the volume of an ellipsoid. In another experiment, subcutaneous tumors were harvested 2 days after the last (3rd) treatment and subjected to immunohistochemical staining to evaluate GFP expression, which indicates successful viral production in tumor tissues. The animal experimental protocol was approved by the Ethics Review Committee for Animal Experimentation of Okayama University. All animal studies were conducted in accordance with the Policy on the Care and Use of the Laboratory Animals, Okayama University.

### CAR blocking assay

HCT116 cells (1.0 × 10^3^ cells/well in a 96-well plate) were treated with Lipo-pTS (0.4 μg/ml) or TelomeScan (10 and 20 multiplicity of infection (MOI)) with or without anti-CAR antibody (8 μg/well) (Merck, Germany) pretreatment. GFP spots were observed with a fluorescence microscope (Olympus IX71) and counted in 3 different randomly-selected microscopic fields (40x) 3 days after treatment.

### Neutralizing antibody assay

HCT116 cells (1.0 × 10^3^ cells/well in a 96-well plate) were treated with Lipo-pTS (0.4 μg/ml) or TelomeScan (80 and 150 MOI) (n = 5) with or without adenovirus type 5-specific antibody (0.5 units/well) (Denka Seiken, Japan) pretreatment. Cell viability was evaluated with the XTT assay 5 days after each treatment.

### Immunogenicity assessment *in vivo*

To evaluate the adenovirus-specific immune response *in vivo*, 5-week-old female BALB/c mice were intravenously treated with Lipo-pTS (10 μg) or TelomeScan (1.0 × 10^9^ plaque-forming units (PFU)) (n = 5) in addition to PBS and liposome twice (day 1 and day 15), and then whole blood was obtained on day 29. H1299 cells, a human non-small cell lung cancer cell line (1.0 × 10^4^ cells/well in a 96-well plate), were treated with TelomeScan (1 MOI) for 3 days together with each serum diluted 1024 times, GFP spots were then observed with the Olympus IX71, and the fluorescence intensity was measured with the BD FACSArray^TM^ Bioanalyzer (BD Biosciences, San Jose, CA).

### Statistical analysis

All data are expressed as means ± SD. Differences between groups were examined for significance with Student’s *t*-test. P values  < 0.05 were considered significant.

## Electronic supplementary material

Supplementary Information

Supplementary Video
