# Supplementary material for: Liposome-encapsulated plasmid DNA of telomerase-specific oncolytic adenovirus with stealth effect on the immune system
Source: Sci Rep. 2017 Oct 26;7:14177. doi: 10.1038/s41598-017-14717-x (PMC5658411; doi:10.1038/s41598-017-14717-x)
Supplement: Supplementary file 1 — Supplementary Information [file 41598_2017_14717_MOESM1_ESM.pdf]

Supplementary Information

Liposome-encapsulated plasmid DNA of telomerase-specific oncolytic adenovirus with stealth effect on the immune system

Katsuyuki Aoyama, Shinji Kuroda, Toshiaki Morihiro, Nobuhiko Kanaya, Tetsushi Kubota, Yoshihiko Kakiuchi, Satoru Kikuchi, Masahiko Nishizaki, Shunsuke Kagawa, Hiroshi Tazawa, Toshiyoshi Fujiwara

Supplementary Fig. 1. Infectivity of Lipo-pTS on H1299 and HT29 cells

Supplementary Fig. 2. Construct of TelomeScan plasmid DNA

Supplementary Fig. 3. Original western blots presented in the main figure (Fig. 2d)

Supplementary Video. Time-lapse imaging of HCT116-RFP cells treated with PBS, Lipo-pGFP, or Lipo-pTS

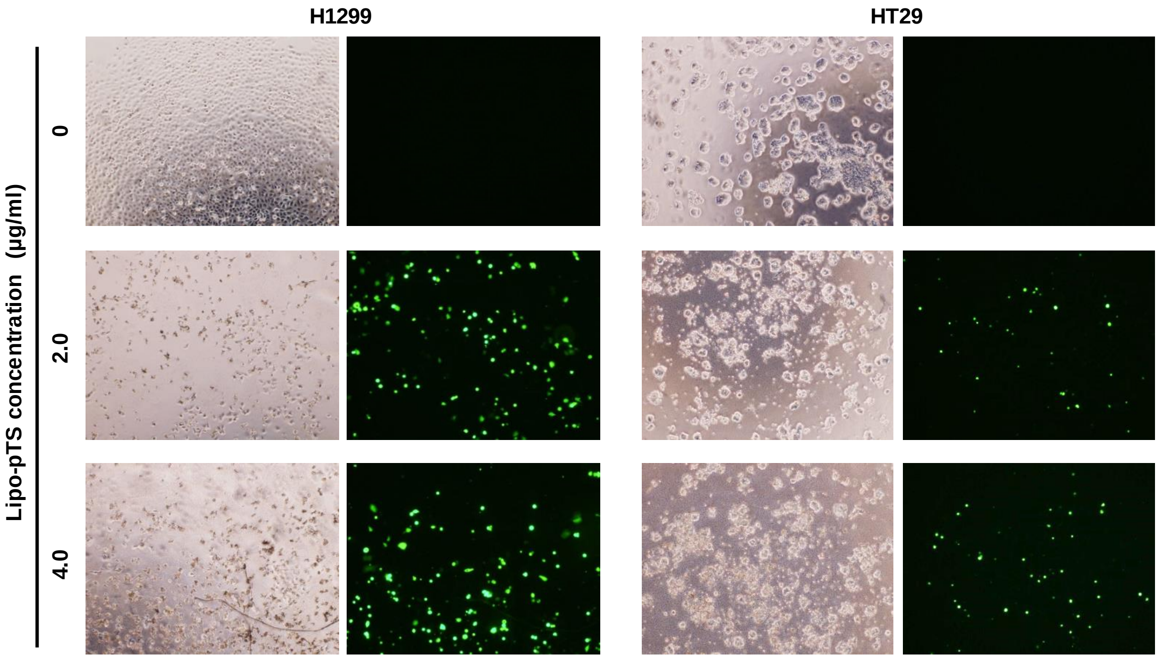

Supplementary Fig. 1. Infectivity of Lipo-pTS on H1299 and HT29 cells

H1299 human non-small cell lung carcinoma cells and HT29 human colon carcinoma cells (5.0 x 10^5 cells/well in a 12-well plate) are treated with 2.0 or 4.0 µg/ml of Lipo-pTS for 3 days, and GFP spots are observed on fluorescence microscopy.

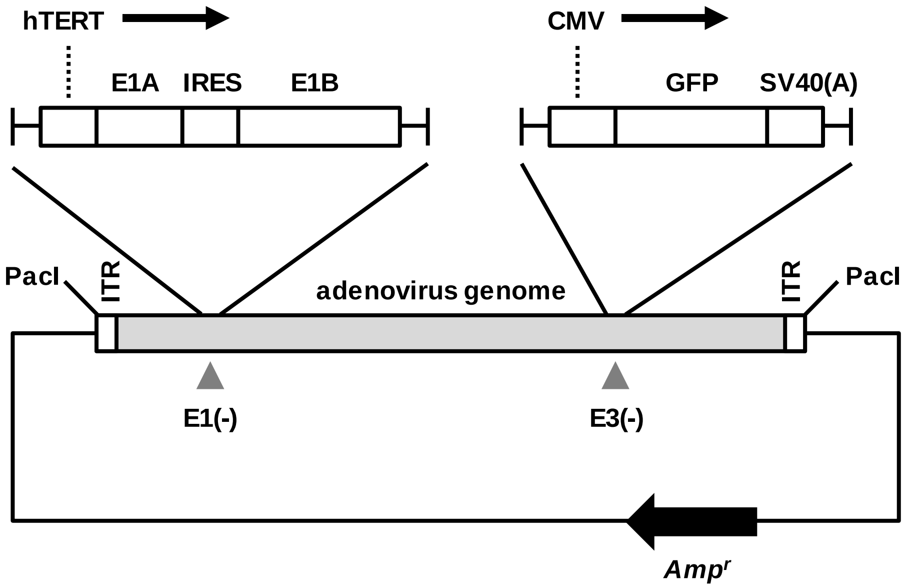

Supplementary Fig. 2. Construct of TelomeScan plasmid DNA

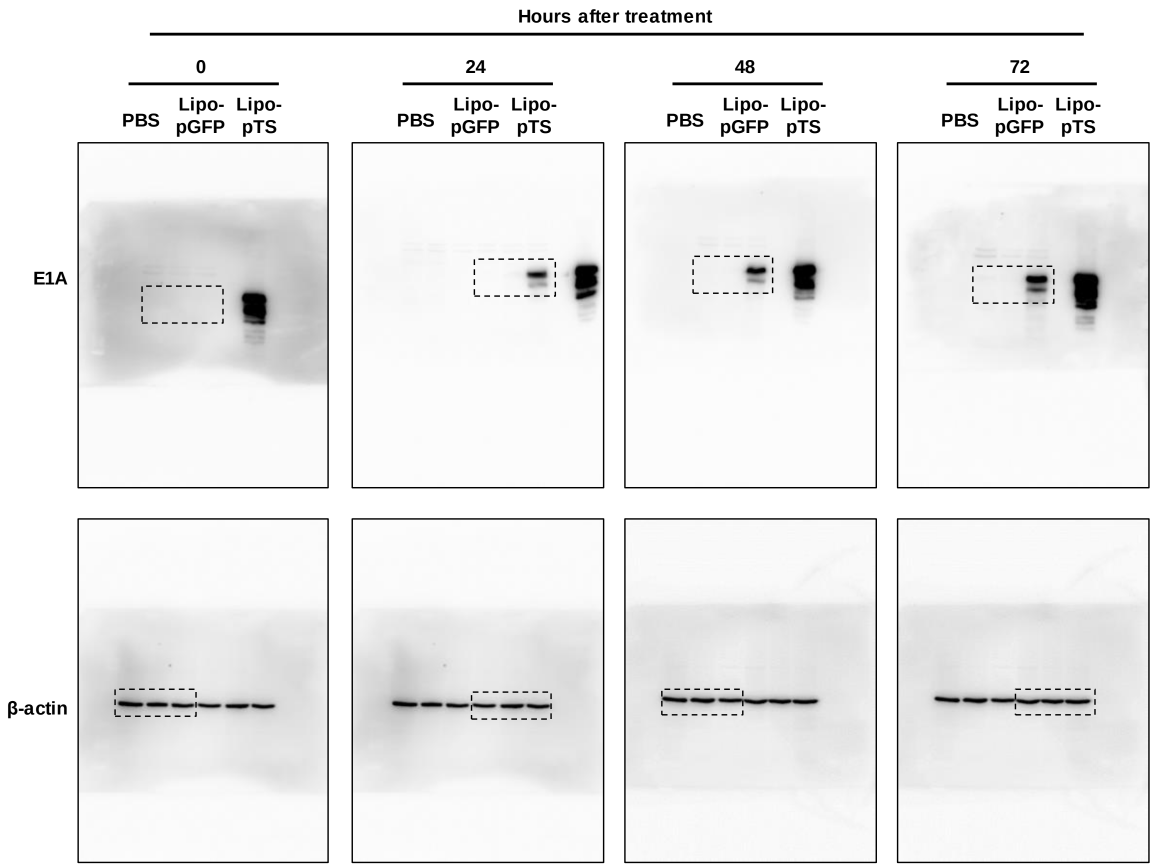

Supplementary Fig. 3. Original western blots presented in the main figure (Fig. 2d)

Supplementary Video. Time-lapse imaging of HCT116-RFP cells treated with PBS, Lipo-pGFP, or Lipo-pTS
